# Comparative Analysis of the Integument Transcriptomes between *stick* Mutant and Wild-Type Silkworms

**DOI:** 10.3390/ijms19103158

**Published:** 2018-10-14

**Authors:** Duan Tan, Hai Hu, Xiaoling Tong, Minjin Han, Songyuan Wu, Xin Ding, Fangyin Dai, Cheng Lu

**Affiliations:** State Key Laboratory of Silkworm Genome Biology, Key Laboratory of Sericultural Biology and Genetic Breeding, Ministry of Agriculture, College of Biotechnology, Southwest University, Chongqing 400715, China; tanzeduan@163.com (D.T.); huhaiswu@163.com (H.H.); xltong@swu.edu.cn (X.T.); hmj251286595@swu.edu.cn (M.H.); fly_jungle@163.com (S.W.); dingx305@163.com (X.D.)

**Keywords:** *Bombyx mori*, *stick* mutant, transcriptome, integument, stiff exoskeleton

## Abstract

In insects, the integument provides mechanical support for the whole body and protects them from infections, physical and chemical injuries, and dehydration. Diversity in integument properties is often related to body shape, behavior, and survival rate. The *stick* (*sk*) silkworm is a spontaneous mutant with a stick-like larval body that is firm to the touch and, thus, less flexible. Analysis of the mechanical properties of the cuticles at day 3 of the fifth instar (L5D3) of *sk* larvae revealed higher storage modulus and lower loss tangent. Transcriptome sequencing identified a total of 19,969 transcripts that were expressed between wild-type Dazao and the *sk* mutant at L5D2, of which 11,596 transcripts were novel and detected in the integument. Differential expression analyses identified 710 upregulated genes and 1009 downregulated genes in the *sk* mutant. Gene Ontology (GO) enrichment analysis indicated that four chitin-binding peritrophin A domain genes and a chitinase gene were upregulated, whereas another four chitin-binding peritrophin A domain genes, a trehalase, and nine antimicrobial peptides were downregulated. Kyoto Encyclopedia of Genes and Genomes (KEGG) analysis indicated that two functional pathways, namely, fructose and mannose metabolism and tyrosine metabolism, were significantly enriched with differentially-expressed transcripts. This study provides a foundation for understanding the molecular mechanisms underlying the development of the stiff exoskeleton in the *sk* mutant.

## 1. Introduction

Insects are the most diverse group of organisms on the planet, representing more than half of all known living species. These organisms exhibit both inter- and intraspecies differences in exoskeleton shapes. Insects have evolved morphologically distinct exoskeleton shapes to match the behavior of their populations and to adapt to specific environments in which they live to survive. For example, stick insects (*Ctenomorphodes chronus*) and leaf insects (*Phyllium* spp.) show dramatically different exoskeleton shapes despite sharing the same body plans. Previous studies have found that insect exoskeleton shapes are controlled by complex gene regulatory networks such as the insulin signaling pathway [1,2,3,4], Hippo pathway [5], *Hox* gene family [6], chitin synthesis and metabolic genes [7,8,9,10,11], cuticle protein genes [12,13], cell cycle-related genes [3], muscle development and differentiation genes [14], and calmodulin genes [15].

The exoskeleton plays an important role in the development and metamorphosis of insects. The structure and mechanical properties of the exoskeleton affect the adaptability of an insect to its immediate environment. For insects, the exoskeleton serves as the base for muscle attachment and the first line of defense from bacteria, fungi, predators, and parasites, as well as environmental chemicals, such as pesticides. Chitin (β (1-4) linked *N*-acetylglucosamine residues), cuticular proteins, resilin, protective cuticular proteins (e.g., cecropin), and cuticular lipids are important and indispensable chemical components of cuticles and play important roles in the development and differentiation of insect exoskeletons. Chitin and cuticular proteins are two of the most important elements of insect exoskeletons.

In the synthesis and metabolic pathway of chitin, trehalose is catalyzed by a series of enzymes (e.g., chitin synthase, CHS) to synthesize chitin, which then cross-links with cuticular proteins to form the extracellular matrix of insects. Meanwhile, the chitin of the extracellular matrix can be degraded into *N*-acetylglucosamine by the corresponding enzymes (e.g., chitinase, CHT) [16]. Two genes encode CHS in most insects. One is CHS-A (encoded by *Chs-1*), which is mainly expressed in epidermal cells and is responsible for integument construction [17,18,19,20]. The other is CHS-B (encoded by *Chs-2*), which is expressed in midgut tissues and is responsible for forming the peritrophic matrix [21,22,23,24,25]. The abnormal expression of *Chs-1* in *Drosophila melanogaster* embryos can lead to cuticle integration defects, resulting in a deformed exoskeleton [26,27]. Meanwhile, knocking down *Chs-1* expression in *Bombyx mori*, *Tribolium castaneum*, and *Locusta* leads to variations in exoskeleton shape, chitin-deficient cuticle, molting failure, and increased mortality [21,25,28]. Chitinases belong to the 18-glycosyl hydrolase family, which is mainly responsible for the degradation of chitin. The number of genes encoding chitinases vary among insect species, ranging from 7 to 22 [29,30]. There are 12, 16, 20 and 22 genes that encode chitinase and chitinase-like genes in *B. mori*, *Drosophila*, *Anopheles gambiae*, and *T. castaneum*, respectively [29,31,32]. The chitinase gene *Cht5* in *T. castaneum* has been shown to be essential for pupal-adult molting, and knocking down its transcription may lead to death [33]. Similarly, RNAi-mediated suppression of *Cht5-1* transcripts in *Locusta migratoria* leads to severe molting defects and lethality [34].

Cuticular proteins are also important components of insect exoskeleton, which fills in the matrix around chitin rods. A large number of cuticle protein genes have been identified in representative insects; *D. melanogaster* and *T. castaneum* harbor more than 100 genes, and *B. mori* and *A. gambiae* have more than 200 cuticular protein genes [35,36,37]. Based on sequence characteristics, cuticle proteins can be divided into several families, including CPR (Rebers and Riddiford motifs) [38], CPF (contains a highly-conserved domain of 44 amino acids), CPFL (CPF-like) [39], CPT (Tweedle gene motif) [12], CPAP (ChtBD2 motif) [40,41], CPG (glycine-rich residue) [36], CPLC (containing low-complexity sequences, including the CPLCA, CPLCG, CPLCP, and CPLCW subfamilies) [37], and apidermin [42]. The absence or abnormal expression of cuticular protein genes may directly result in abnormal or abortive exoskeleton construction. In *D. melanogaster*, *TweedleD* (CPT) null mutations result in stout larvae and pupa [12]. In the absence of *obst-E* (Obstructor-E, belonging to the CPAP cuticular protein family) function, the *D. melanogaster* larval cuticle fails to undergo metamorphosis and ultimately forms a twiggy pupal body [13]. Downregulation of *CRP4*, *CPR18*, and *CPR27* in *T. castaneum* by RNA interference generates adults with shorter, wrinkled, warped, fenestrated, and less rigid elytra compared to the control. Furthermore, adults prematurely died possibly due to dehydration approximately one week after eclosion [43,44]. Hence, cuticular components are considered one of most important factors determining exoskeleton shape in insects.

The silkworm *B. mori* is an economically significant insect. It is also a Lepidoptera model based on its abundant genetic resources and has been used in classical genetic studies for almost 100 years [45]. More than 600 silkworm mutant strains have been obtained and properly preserved in China and Japan, which include approximately 30 mutant strains involving exoskeleton shape variations, thereby offering a valuable resource for investigations of the molecular mechanism underlying shaping insect exoskeleton. To date, only three mutants associated with exoskeleton shape variations have been successfully identified and characterized. The *stony* (*st*) mutant larvae show whole-body stiffness, tightness, and hardness; bulging intersegmental folds; and severe defects in larval adaptability. A previous study showed that *BmorCPR2* is responsible for the silkworm *st* mutant [46]. Moreover, a fast-evolving gene, *BmorCPH24*, which encodes a cuticular protein with low-complexity sequence, is the gene responsible for the *Bomboo* (*Bo*) mutant, which exhibits a dilated thorax and a slim but hard abdomen; the segmental boundaries bulge outward from the body, and the main area of each segment is narrow and constricted [47]. A serine protease homologue *Bmscaface* can induce an abnormal exoskeleton shape in mutant silkworm larvae called *tubby* (*tub*), which exhibits an abnormally short, fat exoskeleton, and also breadthwise has expanded to form a fusiform exoskeleton [48].

**Figure 1:**
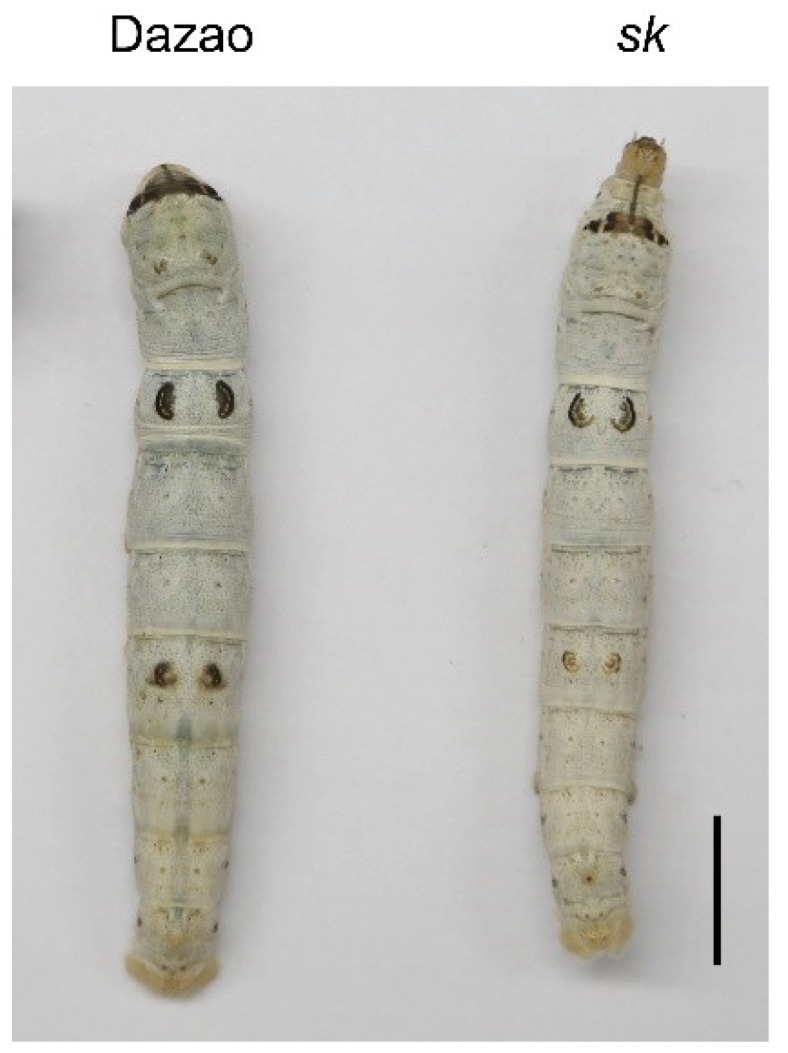
Phenotypes of the *stick* mutant (*sk*/*sk*) and wild-type strain (Dazao) at L5D3. The *sk* mutant larvae exhibited a stick-like slender, firm larval body, which was particularly observed in L5D3 larvae. Scale bar: 1 cm.

The *stick* (*sk*) mutant, a spontaneous autosomal recessive mutation, was first reported in 1927 and was subsequently preserved in China and Japan [49]. The *sk* mutant exhibits a stick-like exoskeleton with a slender, less flexible, and firm-to-the-touch larval body compared to wild-type Dazao (Figure 1) at L5D3. Classical genetic analysis demonstrated that the *sk* mutant is a single-locus mutation linked to chromosome 4 [49]. Elucidation of the molecular basis of the *sk* mutant can improve our understanding of insect exoskeleton development. Thus, the present study conducted a comparative analysis of transcriptomes between the *sk* mutant and wild-type Dazao to identify candidate genes involved in generation of the *sk* mutant.

## 2. Results

### 2.1. Comparison of the Mechanical Properties of Cuticle between the Wild-Type and sk Mutant

**Figure 2:**
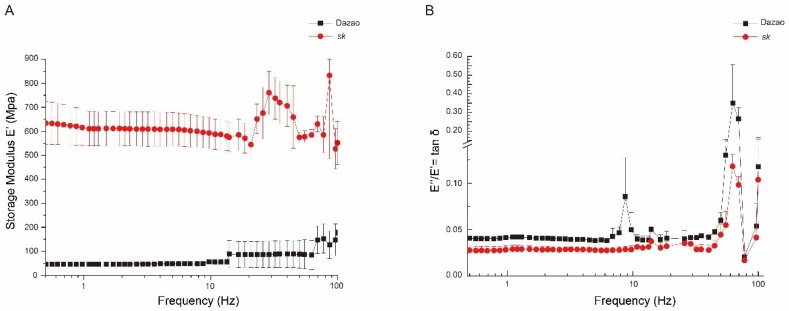
Mechanical properties of day 3 of the fifth instar larval cuticles between Dazao and *sk* mutant. (**A**) Storage modulus (E′) of L5D3 larval cuticles of wild-type Dazao and *sk* mutant under frequency scanning (*n =* 3). (**B**) tanδ (E′′/E′) of L5D3 larval cuticles of wild-type Dazao and *sk* mutant under frequency scanning (*n =* 3).

Insect bodies are coated by cuticle (exoskeleton), which is a matrix composed of various proteins and the polysaccharide chitin. In *D. melanogaster*, the larval cuticle confers oriented contractility/expandability to determine its pupal exoskeleton shape for mechanical control [13]. Analogously, the *sk* mutant shows a constrictive stick-like larval body, suggesting that cuticle ductibility is insufficient. Here, we investigated the mechanical properties of the cuticle between the wild-type and *sk* mutant at L5D3 using a dynamic mechanical analyzer (DMA Q800). The primary parameters for evaluating dynamic mechanical properties of the samples include modulus of elasticity (E′), loss modulus (E′′), and loss tangent (E′′/E′ ratio, termed “mechanical loss factor”), respectively. First, E′ is presented as the ratio of normal stress to normal strain during the elastic deformation stage of the samples. Second, E′′ represents energy loss caused by viscous deformation when the sample is deformed. Third, the E′′/E′ ratio, as represented by tanδ, is assessed via dynamic mechanical analysis (DMA), which provides information associated with the inner molecular structures [50]. Our results showed a reduction in E′ in both types of cuticles with decreasing frequency, whereas that of the *sk* mutant cuticles were higher than that of wild-type cuticles at all times (Figure 2A). Nevertheless, a decrease in tanδ was observed in the cuticles of both wild-type and *sk* mutant with decreasing frequency, and that of the wild-type was consistently higher than that of *sk* mutant (Figure 2B). These findings indicate that the exoskeleton of the *sk* mutant exhibits a higher degree of stiffness and less damping oscillation compared to the wild-type.

### 2.2. Transcriptomic Sequence Analysis and Detection of Differentially-Expressed Genes (DEGs)

The *sk* mutant phenotype that includes a stiff exoskeleton appears on L5D3. The *sk* mutants and wild-type strain Dazao of the same developmental stages were selected at day 2 of the fifth instar (L5D2). The integument transcriptomes of the two groups were individually sequenced, and six mRNA libraries were generated from the *sk* mutant and wild-type (three repeats for each group). Approximately 29.3–36.3 million raw reads were obtained. After quality filtering, the remaining 28.3–35.2 million clean reads were mapped to the silkworm genome. Subsequently, 85.1–89.7% of the total clean reads were uniquely mapped to the *B. mori* genome, covering 19,969 predicted genes, which include 8373 predicted genes in SilkDB (http://www.silkdb.org/silkdb/, accessed on 2 January 2018), and 11,596 novel transcripts. An average of 73.8% of the tags of cross-intron reads were well mapped to known exons, and 26.2% of those were located in predicted intronic and intergenic regions. The major characteristics of the six libraries and novel transcripts are summarized in Supplementary Tables S1 and S2. The raw reads of these RNA-Seq libraries were deposited in the Sequence Read Archive (SRA) database of NCBI as accession number SRP160891.

**Figure 3:**
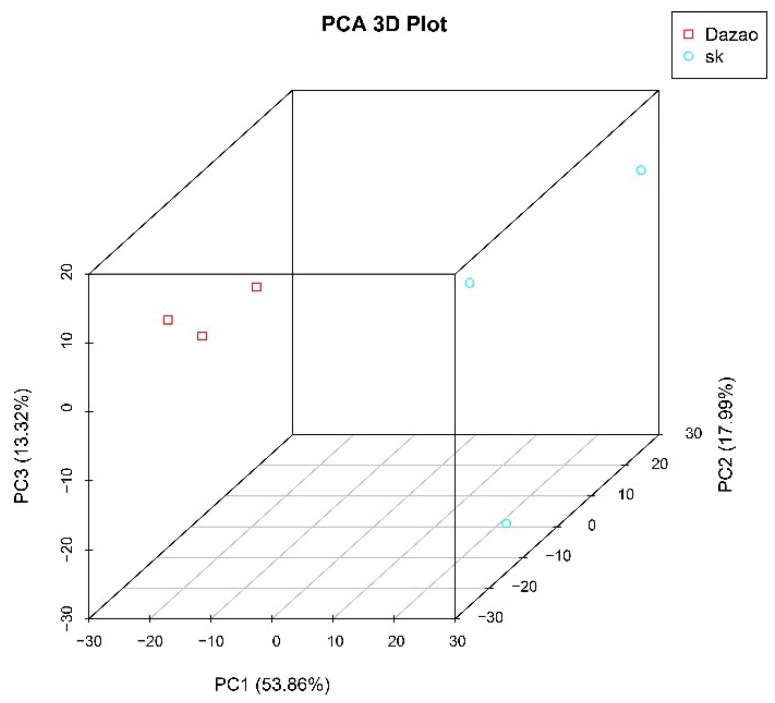
Principal component analysis (PCA) for all RNA-Seq sample. Dazao are represented by the red dots, the *sk* mutants are indicated by the blue dots.

Principal component analysis (PCA) showed that 85.2% of the transcriptional differences between the wild-type and *sk* mutant were accounted for by three PCA components. Among the three components clustered the samples associated with the same silkworm strain, whereas the samples between the wild-type and *sk* mutant were dispersively arranged (Figure 3).

**Figure 4:**
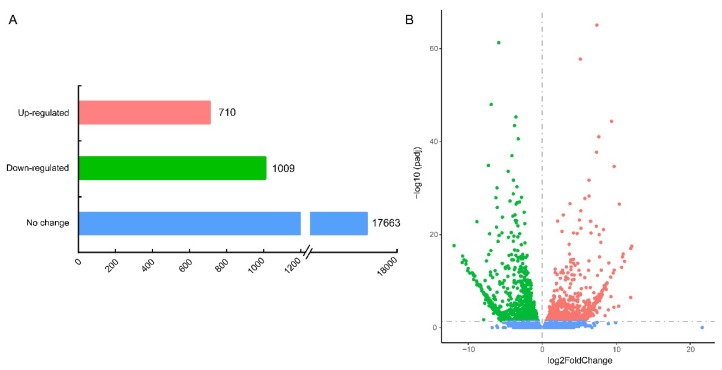
Expression levels between *sk* and wild-type Dazao. (**A**) The number of DEGs and non-regulated genes between *sk* compared to the wild-type Dazao. (**B**) Volcano plot of log2 fold-change against negative log10 of FDR value (*sk* mutant versus wild-type Dazao). Red represents upregulated genes, green represents downregulated genes, and blue represents unaltered genes in the *sk* mutant compared to the wild-type Dazao under the criteria of FDR ≤ 0.05.

To investigate the exoskeleton shape-related genes, comparative analysis of the normalized data on the *sk* mutant vs. wild-type was performed to identify DEGs. Under the multiple hypotheses test and the corrected *p* value (FDR) ≤ 0.05, a total of 1719 DEGs were detected between the *sk* mutant and the wild-type, including 710 upregulated and 1009 downregulated genes in the *sk* mutant, and the distribution of the DEGs is presented in Figure 4 and Supplementary Table S3.

### 2.3. Validation of RNA-Seq Data by qRT-PCR

**Figure 5:**
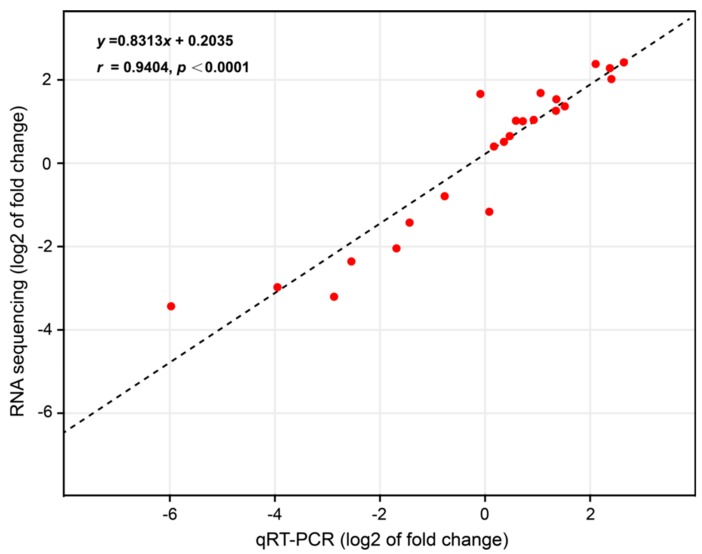
The Pearson correlation analysis of RNA sequencing data and qRT-PCR results for the 20 selected DEGs and 1 non-DEG between *sk* and Dazao. Each red point denotes a value of fold change of expression level in the *sk* mutant compared to the wild-type Dazao. The fold-change values were transformed by log10.

To confirm the reliability of the RNA-Seq data generated in this study, 20 DEGs with different expression profiles and *BGIBMGA000563* (also known as tyrosine hydroxylase, *TH*) with no significant differences in expression were selected and used in qRT-PCR analysis. Our results showed a high level of consistency between the RNA-Seq data and qRT-PCR (*r* = 0.9404, *p* < 0.0001, Figure 5). For each detected gene, the expression level in the RNA-Seq data showed a consistent expression pattern compared to the results of qRT-PCR, indicating that our transcriptome data are highly reliable.

### 2.4. GO and KEGG Pathway Enrichment Analysis of DEGs

**Figure 6:**
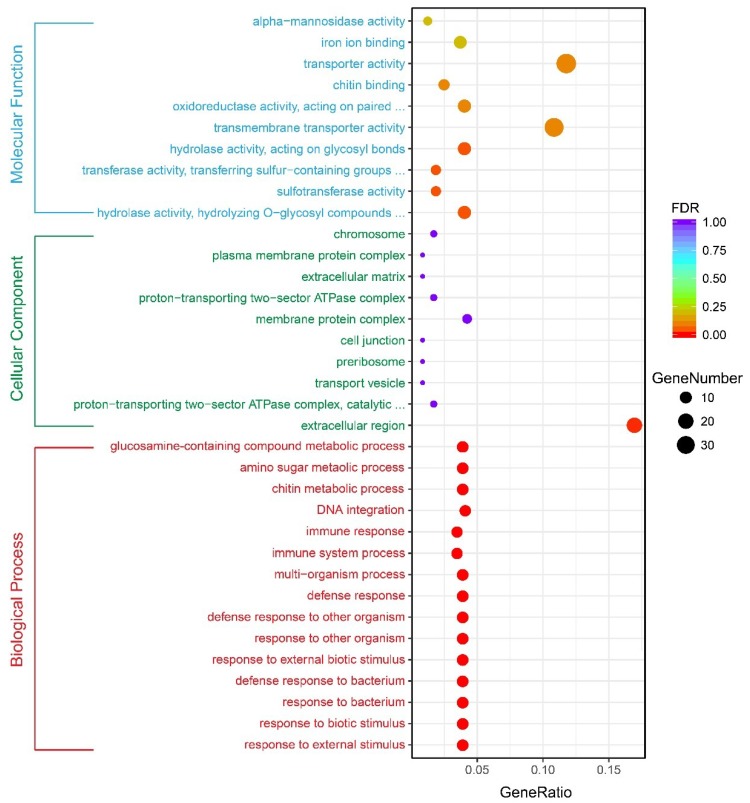
Gene ontology (GO) analyses of DEGs. The *x*-axis represents the number and percent of unigenes mapped to the indicated GO term. The *y*-axis is gene functional classification of GO.

To further investigate the DEGs involved in larval exoskeleton shape, functional annotation was performed based on GO categories. GO enrichment analysis identified GO terms that were related to the DEGs of the *sk* mutant vs. wild-type (Figure 6). Among these, 19 genes were also significantly mapped to molecular functions, 20 genes were significantly mapped to cellular components, and 28 genes were significantly mapped to biological processes (FDR < 0.05, Table 1). Of the molecular function-related genes, these were mainly involved in hydrolase activity (hydrolyzing *O*-glycosyl compounds), sulfotransferase activity, transferase activity (transferring sulfur-containing groups), and hydrolase activity (acting on glycosyl bonds). Most of the cellular component-related genes were only involved in extracellular regions. However, most of the biological process-related genes were involved in response to external stimulus, response to biotic stimulus, response to bacterium, defense response to bacterium, response to external biotic stimulus, response to other organism, defense response to other organism, defense response, multi-organism process, immune system process, immune response, DNA integration, chitin metabolic process, amino sugar metabolic process, and glucosamine-containing compound metabolic process.

**Figure 7:**
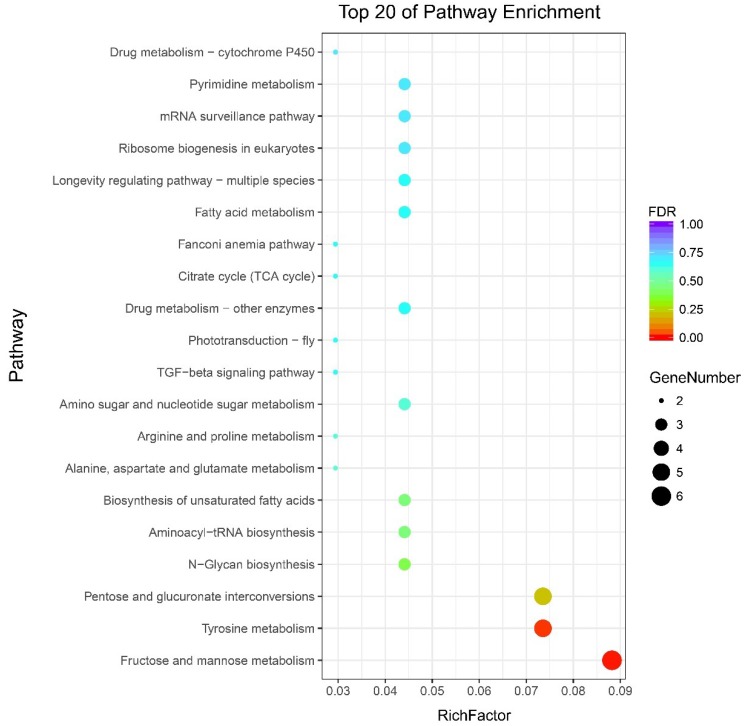
Scatterplot of enriched KEGG analyses for DEGs. The rich factor indicates the ratio of DEG numbers annotated in this pathway group to total gene numbers annotated in this pathway group. The larger the rich factor, the larger degree of pathway enrichment. The FDR ≤ 0.05 means significant pathway enrichment.

All DEG genes were subjected to Kyoto Encyclopedia of Genes and Genomes (KEGG) annotation and enrichment analysis, which revealed that 1677 of the 1719 DEGs could be assigned to 53 KEGG pathways (Supplementary Table S4). The top 20 enrichment KEGG terms were drawn as a scatter plot (Figure 7). The enriched pathways could be divided into several functional aspects, including carbohydrate metabolism, amino acid metabolism, pentose and glucuronate interconversions, as well as biosynthesis of other secondary metabolites. The results show that only two KEGG pathways were significantly enriched. The only two metabolism pathways, namely, fructose and mannose metabolism and tyrosine metabolism were significantly enriched (Supplementary Table S5). In the functional category of fructose and mannose metabolism, five genes, including *BGIBMGA014323* (mitochondrial enolase superfamily member 1-like), *BGIBMGA008433* (GDP-mannose 4,6 dehydratase), *novel.11049* (zinc-binding dehydrogenase/alcohol dehydrogenase GroES-like domain), *BGIBMGA005687* (GDP-mannose 4,6 dehydratase), and *BGIBMGA006473* (mannose-6-phosphate isomerase) were significantly upregulated in the *sk* mutant. However, only one gene, *BGIBMGA012831* (aldo-keto reductase AKR2E4 isoform X1), was downregulated. In addition, five genes enriched the tyrosine metabolism term, of which four belonged to the Yellow family, including *novel.3420* (major royal jelly protein), *novel.12441* (major royal jelly protein), *novel.12443* (l-dopachrome tautomerase yellow-f-like), and *BGIBMGA014026* (Yellow8). *Novel.3420*, *novel.12441*, and *novel.12443* were upregulated in the *sk* mutant, whereas *BGIBMGA014026* was downregulated. The remaining gene was *BGIBMGA002087* (macrophage migration inhibitory factor) and was downregulated in the *sk* mutant (Supplementary Table S5). These annotations provide novel insights into studying extraordinary pathways, processes, and functions involved in silkworm larval exoskeleton shape.

### 2.5. DEGs Specifically Related to Chitin Binding and Chitin Metabolism

The cuticle of insect is composed of the epicuticle, exocuticle, and endocuticle. Chemical composition of cuticle consists of chitin, cuticular proteins (including stage-specific differences in cuticular proteins), resilin, protective cuticular proteins (e.g., cecropin), and cuticular lipids. A list of nine DEGs related to chitin metabolism and chitin binding is presented in Table 2. Functional annotation of DEGs identified two groups, namely, chitin-binding peritrophin A domain protein genes and chitinase. The chitin-binding peritrophin A domain protein genes consisted of eight DEGs, including *novel.3284*, *novel.4995*, *novel.6300*, *novel.8828*, *novel.311*, *novel.5689*, *novel.6971*, and *BGIBMGA006382*; the first four genes were clearly upregulated in the *sk* mutant, whereas the latter four genes were downregulated. *BGIBMGA008709*, which encodes chitinase, was significantly upregulated in the *sk* mutant. Chitin is one of the major constituents of cuticle, a biopolymer of *N*-acetyl-β-d-glucosamine residues that are held together by β-(1-4)-glycosidic linkages. In insect cuticle, cuticular proteins often fill in the matrix around chitin rods. In hard cuticles, cuticular proteins, chitin, phenolic, and quinone compounds form covalent bonds and cross-link with each other to constitute a very hard, rigid structure. Nevertheless, in very soft cuticles, there is some degree of stabilization, yet probably only involve relatively few cross-links. Chitinase is one of components of molting fluid and digests chitin in old endocuticle. Chitinase is an endoenzyme that randomly attacks the chitin chain by internal hydrolysis. These results implied that chitin-binding peritrophin A domain protein genes and chitinase may play an important role in the construction of larval exoskeleton in silkworm and is associated with the mechanical properties of larval cuticle.

### 2.6. DEGs Related to Hydrolase and Transferase

GO analysis showed that hydrolase was highly significantly enriched. Hydrolase is commonly used as a biochemical catalyst that utilizes water to break chemical bonds. Common hydrolases are esterases, which include glycosidases, lipases, phosphatases, peptidases, and nucleosidases. A list of 12 DEGs related to hydrolase is provided in Table 2. These DEGs consist of six groups: glycosyl hydrolase (also called glycoside hydrolase), mannosidase, trehalase, glucosidase, destabilase, and galactosidase. For glycosyl hydrolase, six DEGs encoding glycosyl hydrolase (families 1, 31 and 38), namely, *novel.2031*, *novel.12594*, *novel.12616*, *BGIBMGA013995*, *novel.1424*, and *novel.15227*, were downregulated in *sk*; these catalyze the hydrolysis of glycosidic bonds in complex sugars [51,52]. Together with glycosyltransferases, glycosyl hydrolase forms the major catalytic machinery for the synthesis and breakage of glycosidic bonds. For mannosidase, two genes, namely, *BGIBMGA002426* and *BGIBMGA002486*, which encode α-1,2-mannosidase, were downregulated in *sk*. Mannosidase is an enzyme that hydrolyses mannose and includes two types, namely, α-mannosidase and β-mannosidase. Another DEG, *BGIBMGA005665*, encodes trehalase 1B, was also clearly downregulated. Trehalase is located on the brush border of the small intestine and catalyzes the conversion of trehalose to glucose [53,54,55]. Three DEGs encoding β-glucosidase, destabilase, and α galactosidase A, namely, *BGIBMGA010811*, *novel.6630*, and *novel.8493*, were upregulated in *sk*. Most of the hydrolase-related DEGs were downregulated, and only three of these genes were upregulated in *sk*. Together, these results implied reduced hydrolase activity in *sk* but not in Dazao.

Sulfotransferases are transferase enzymes that catalyze the transfer of a sulfo group from a donor molecule to an acceptor alcohol or amine [56]. Generally, the reactive groups for a sulfonation via sulfotransferases may be part of a protein, lipid, carbohydrate, or steroid [57]. This reaction is widely observed from bacteria to humans and indicate that sulfotransferases play important roles in development, differentiation, and homeostasis [58]. Four DEGs encoding sulfotransferases, namely, *novel.2592*, *novel.6336*, *novel.15388*, and *BGIBMGA007552*, were downregulation in *sk*, whereas two other homologous genes, namely, *novel.1630* and *BGIBMGA010842*, were upregulated in *sk*.

### 2.7. DEGs Related to Defense and Immune Responses

Among the important constituents of cuticle are the protective cuticular proteins that play crucial roles in protecting against invading bacteria or other organisms. The protective cuticular proteins, also called antimicrobial peptides such as cecropin, attacin, moricin, lebocin, enbocin, and gloverin have been isolated from *B. mori* [59,60,61,62,63,64]. A total of nine DEGs that encode antimicrobial peptides were downregulated in the *sk* mutant compared to the wild-type Dazao. Among these, six DEGs encode cecropin, including *BGIBMGA006280*, *BGIBMGA014285*, *BGIBMGA000023*, *BGIBMGA000036*, *BGIBMGA000021*, and *BGIBMGA000017*. Another two DEGs encode enbocin, including *BGIBMGA000039* and *BGIBMGA000018*, and one DEG encodes moricin, *BGIBMGA011495*. Overall, these results imply that some components may be defective in the *sk* larval cuticle, leading to changes in exoskeleton shape and a decrease in the ability to resist eliminating invaders.

## 3. Discussion

The present study conducted RNA-seq to analyze the transcriptomes of the integument and identified genes that are expressed in the integument that were predicted to have function in larval exoskeleton shape formation.

### 3.1. The Cuticle of sk Mutants Shows a Higher Degree of Stiffness and Less Damping Oscillation Compared to the Wild-Type

The stretching mode of DMA often provides an excellent strategy to measure the dynamic mechanical properties of film-like materials. The fifth larval silkworm cuticles are wide and thin and hence can be treated as film-like material to analyze the mechanical properties between wild-type and *sk* mutant strains. The cuticle is one of the important components of insect exoskeleton, which provides support for the whole body and determines the exoskeleton shape. Therefore, information on the mechanical properties of differentially-expressed genes in mutants with abnormal exoskeleton shapes related to cuticle formation can improve our understanding of the mechanism underlying differential larval exoskeleton shapes in insects. DMA analysis of E′ offers a method for measuring stiffness of materials [65,66,67]. Our results demonstrated that the E′ of the *sk* mutant is higher than that of the wild-type Dazao strain, suggesting that the cuticle of the *sk* is stiffer (Figure 2A). On the other hand, tanδ represents the damping characteristics of materials [65,66,67]. Higher tanδ values were observed in the wild-type, which denoted that the damping characteristics of the wild-type were better compared to those of the *sk* mutant (Figure 2B). Better damping characteristics of materials allows the dissipation of energy absorbed in the form of heat, consequently reducing their amplitude and thereby resulting in the damping of oscillations. These results indicate that the cuticles of the wild-type silkworm are better at damping oscillation compared to those of the *sk* mutant, particularly when these are subjected to external forces. The abnormal larval exoskeleton shape of the *sk* mutant leads to cuticles with less storage modulus and damping factor, thereby reducing their capacity to adapt to the environment.

### 3.2. Cuticular Proteins and Chitinase Are Associated with Larval Exoskeleton Shape Formation

Cuticular proteins are major components of the insect cuticle, and more than 200 cuticular protein genes have been described in the *B. mori* based on their distinct domain sequence characteristics [36,68]. High amounts of chitinases are present in molting fluid and represent one type of endoenzyme that randomly attacks chitin chains by internal hydrolysis, playing essential roles in the molting and formation of new cuticles in insects.

Previous studies have shown that the functions of some cuticular proteins have been associated with insect exoskeleton shape, immunity, and wing patterns. However, the molecular functions of most of these are unclear. Compared to the wild-type Dazao, four cuticular proteins were found to be extremely significantly downregulated, including *novel.3284*, *novel.4995*, *novel.6300*, and *novel.8828*. On the contrary, four other cuticular proteins were found to be significantly upregulated, including *novel.311*, *novel.5689*, *novel.6971*, and *BGIBMGA006382* in *sk* mutants (Table 2). All of the eight cuticular protein genes possessed the same peritrophin A-type chitin-binding domain (ChtBD2). Among these, seven are novel transcripts, and only one was well identified, namely, *BmCPAP1-D* [69]. The ChtBD2-type of the pfam01607 family has cysteine-containing type-2 chitin-binding domain (CBD), a six-cysteine motif found in insect CBDs from both the cuticle and the peritrophic membrane (PM), which was initially thought to be specific to proteins in the PM [70]. In insect, the ChtBD2-containing CBDs were mainly divided into three families, including peritrophic matrix proteins (PMPs), cuticle proteins analogous to peritrophins (CPAPs) containing either a single CBD, namely CPAP1 family, and CPAP3 containing three CBDs (CPAP3 family), and that without other identifiable conserved domains. In addition to the above representative three families of chitin-binding proteins, some chitin metabolism enzymes related to the PM and/or cuticle also contain ChtBD2 domains such as the chitinases and chitin deacetylases [16,29,71]. In *D. melanogaster*, *CPAP3* genes have previously been named “*gasp*” or “*obstructor*” [72,73].

Functional characterization of *CPAP* genes by RNA interference in *T. castaneum* revealed that many of these genes are essential and have non-redundant functions in maintaining the structural integrity of the cuticle in different tissues of insects and at different developmental periods [41]. Loss of the *obstructor-A* (*obst-A*) gene in *D. melanogaster* causes early lethality in larvae and reduction in body size. In addition, *obst-A* is required for epithelial extracellular matrix dynamics, cuticle integrity, and tracheal tubulogenesis [74]. Furthermore, in the absence of *obstructor-E* gene, the *D. melanogaster* larval cuticle fails to undergo metamorphic shape change and finally turns into a twiggy pupal body [13]. In the *B. mori*, *six BmCPAP3s* (including *BmCPAP3-A1*, *BmCPAP3-A2*, *BmCPAP3-B*, *BmCPAP3-C*, *BmCPAP3-D1*, and *BmCPAP3-D2*) demonstrated similar binding abilities toward crystalline chitin and colloidal chitin, which were found to be abundant in molting fluid [75]. In this study, *novel.3284*, *novel.4995*, *novel.6300*, and *novel.8828* were significantly downregulated and *novel.311*, *novel.5689*, *novel.6971*, and *BmCPAP1-D* were upregulated in the *sk* mutant when compared to the wild-type Dazao. Additionally, *BGIBMGA008709*, which encodes a chitinase, was upregulated in the *sk* mutants. It is speculated that such changes contribute to the disruption of cuticle formation or cross-linking between cuticular proteins and chitin, resulting in abnormal exoskeleton shape. Moreover, the phenotypic contribution of these genes requires further verification.

### 3.3. Downregulated Genes Related to Trehalose Metabolism

Trehalose is a non-reducing disaccharide that is formed with two glucose molecules joined together by a glycosidic α-(1-1) bond. It has been isolated from certain species of fungi, bacteria, archaea, plants, and invertebrates, and is a major blood sugar in arthropods and fuels flight in insects [76]. Trehalose is well known for its protective ability, stability, and low reactivity because it can withstand heating to 100 °C between pH 3.5–10.0 for 24 h [77]. Trehalase cleaves trehalose into two glucose residues [78]. Trehalose is the first enzyme that is involved in chitin synthesis [16]. Initially, trehalase hydrolyzes trehalose in to β-d-glucose, and hexokinase catalyzes glucose into glucose-6-phosphate. Subsequently, after a cascade of steps of catalyzed biosynthetic reactions associated with isomerase, aminotransferase, mutase, and pyrophosphorylase, the final product UDP-N-acetylglucosamine is generated. Finally, chitin synthase converts UDP-N-acetylglucosamine into chitin [79]. Chitin functions as very tiny, but mechanically strong, scaffold material that is strongly associated with cuticle proteins and invariably determines the mechanical properties of insect cuticle. In this study, *BGIBMGA005665*, which encodes trehalase 1B, was significantly downregulated in the *sk* mutant. We hypothesize that the downregulation of the trehalase 1B gene leads to a deficiency in chitin synthesis in the larvae, thereby resulting in an insufficiency of raw materials for the formation of larval integument.

### 3.4. Less Active Antimicrobial Peptides in sk Mutants

Antimicrobial peptides are ubiquitous defensive proteins of the innate immune system in insects against invading pathogenic microorganisms. In the silkworm, 40 antimicrobial peptides have been reported, including seven families such as cecropin, moricin, gloverin, attacin, enbocin, lebocin, and defensin, based on their sequence similarity [80]. According to the structural characteristics of the peptide chain, these seven families can be divided to three categories: (1) a linear antimicrobial peptide having an α-helical structure and lacking cysteine, including cecropin, moricin, and enbocin; (2) a constitutive antimicrobial peptide rich in proline and/or glycine, such as gloverin, attacin, and leocin; and (3) cysteine-rich cyclic antimicrobial peptide including defensin.

We observed a marked decrease in the expression of nine *B. mori* genes that encode linear antimicrobial peptides consisting of an α-helical structure and lacking cysteines, including six cecropins, two enbocins, and one moricin in the *sk* integument (Table 2). Silkworm cecropins are a large family with 12 members that can be divided into six subfamilies, namely, cecropins A to E [81,82], which are often arranged on the chromosome in tandem, except for cecropin A. Cecropin possesses powerful antibacterial activity against diverse pathogens, including gram-negative and gram-positive bacteria [83,84,85], fungi [86], viruses [87], tumors [88,89], and parasites [90]. Enbocin and moricin also have powerful antibacterial activity against gram-negative and gram-positive bacteria, with moricin showing stronger activity than cecropin [60,91,92]. Although the function of antimicrobial peptides participating in larval exoskeleton shape formation in silkworms is unknown, it is a protective component of cuticles, which establish the integrity of insect exoskeletons during development.

## 4. Materials and Methods 

### 4.1. Silkworm Strains and Tissue Collection

The *stick* (*sk*) mutant silkworm strain and the wild-type strain Dazao were obtained from the Silkworm Gene Bank of Southwest University, Chongqing, China. All larvae were reared with fresh mulberry leaves under 25 °C and 80% relative humidity, with a photoperiod of 12 h light: 12 h dark. The larval integuments were collected at L5D2 on ice without other tissues including head, appendages, fat body, trachea, and somatic muscles. For each sample, the mixture of integuments of three individuals were pooled as one sample. Each integument was washed quickly with 0.01 M phosphate buffered saline, blotted on filter paper, and then stored at −80 °C until RNA extraction.

### 4.2. Mechanical Properties of Cuticles

The mechanical properties of the cuticles of 5L3D larvae were investigated using a DMAQ800 Dynamic Mechanical Analyzer. The cuticles of 5L3D larvae from the wild-type and *sk* mutants were collected on ice 0.01 M phosphate-buffered saline, and the other tissues, including the head, appendages, fat body, trachea, somatic muscles, and epidermal cells were carefully stripped. The cuticles were trimmed to a 1.0 cm × 2.5 cm rectangle shape to verify that the materials tested were of a similar size, and sourced from the second abdominal segment (segment of crescents) to the fifth abdominal segment (segment of star spot). Materials were stored in ice 0.01 M phosphate-buffered saline and incubated at 25 °C for 2 h before mechanical testing, and then fixed between the two grips of analyzer. E′, E′′, and tanδ = E′/E′′ were determined using the film-stretching mode at 0.1% strain (at this level of strain, the cuticles would not be torn apart) and scanned using frequencies ranging from 100 Hz to 0.1 Hz.

### 4.3. RNA Extraction and RNA-Seq

The total RNA of integument samples from *sk* mutant and wild-type Dazao strain was extracted using TRIzol^®^ reagent (Invitrogen, Carlsbad, CA, USA) according to manufacturer’s protocol. The 1% agarose gels were used to monitor the RNA contamination and degradation. By using a NanoPhotometer^®^ spectrophotometer (IMPLEN, Los Angeles, CA, USA), we checked the total RNA purity of each sample. Then, the Qubit^®^ RNA Assay Kit in Qubit^®^2.0 Fluorometer (Life Technologies, Carlsbad, CA, USA) and the RNA Nano 6000 Assay Kit of the Bioanalyzer 2100 system (Agilent Technologies, Palo Alto, CA, USA) were used to determine the RNA concentration and integrity, respectively. For RNA sample preparations, 3 µg total RNA of each sample was draw as input material. Under the direction of manufacturer, the sequencing libraries was prepared by using NEBNext^®^ UltraTM RNA Library Prep Kit and index codes were added to attribute sequences to each sample. We extracted the mRNA of each sample from the total RNA by using poly-T oligo-attached magnetic beads. Then, the mRNA was fragmented by the divalent cation with an elevated temperature in NEBNext First Strand Synthesis Reaction Buffer (5×). The First strand and the second-strand cDNA were subsequently synthesized using random hexamer primer and M-MuLV Reverse Transcriptase (RNase H-) and DNA polymerase I and RNase H, respectively. Sequencing adaptors were ligated to cDNA fragments of preferentially 250~300 bp in length from each cDNA library, the ligated the library fragments were purified with AMPure XP system (Beckman Coulter, Beverly, USA) and enriched by PCR amplification. After cluster generation, the library preparations were sequenced on an Illumina HiSeq 2000 platform (Illumina, USA) and 125-bp/150-bp paired-end reads were generated.

### 4.4. Novel Transcripts Prediction and Normalization of Gene Expression Levels and DEGs Screening

Novel transcripts prediction was conducted using StringTie (v1.3.3b) [93] using a reference-based approach that was based on assembling the mapped reads of each sample. StringTie assembles and quantitates full-length transcripts representing multiple splice variants for each gene locus based on a novel network flow algorithm as well as an optional de novo assembly step.

Raw data (raw reads) of fastq format were first processed through in-house Perl scripts. In this step, clean data (clean reads) were obtained by removing reads containing adapter, reads containing ploy-N, and low-quality reads from the raw data. Meanwhile, Q20, Q30, and GC content of the clean reads were calculated. All the downstream analyses were based on the high-quality clean reads. The reference genome and gene model annotation files of *B. mori* were downloaded from the Silkworm Genome Database (SilkDB; http://www.silkdb.org/silkdb/, 2 January 2018). All clean reads were well mapped to the silkworm genome and gene reference sequences using Hisat2 (version 2.0.5). The FeatureCounts v1.5.0-p3 was used to count the reads numbers mapped to each gene. The expression level of each gene was measured by fragments per kilobase of transcript sequence per million base pairs sequenced (FPKM), which also considers the effect of sequencing depth and gene length for the reads count at the same time. The FPKM of each gene was calculated based on the length of the gene and reads count mapped to this gene.

After obtaining the normalized gene expression level, differential expression analysis was performed using the DESeq2 R package (1.16.1) to screen the DEGs between the *sk* mutant and wild-type Dazao strain. DESeq2 provide statistical routines for determining differential expression in digital gene expression data using a model based on the negative binomial distribution. The resulting *p*-values were adjusted using the Benjamini and Hochberg’s approach for controlling the false discovery rate. Genes with an adjusted *p*-value ≤ 0.05 found by DESeq2 were designated as DEGs.

### 4.5. GO and KEGG Enrichment Analysis of DEGs

The clusterProfiler R package was used for gene length bias correction and the GO enrichment analysis of DEGs. 0.05 (corrected *p* value) was selected as the threshold to judge whether the GO terms were significantly enriched by differential expressed genes. Three independent GO categorization hierarchies were present, including molecular function, cellular component, and biological process. Besides, we also implemented the clusterProfiler R package to test the statistical enriched KEGG pathways of differential expression genes, the pathway with *p* value ≤ 0.05 was considered as a significant enriched pathway. Lastly the log transformed FPKM values with all sequenced genes of each sample was used for principal component analysis (PCA) and Pearson correlation coefficient analysis in the R package.

### 4.6. Quantitative Real-Time Reverse Transcription PCR

The expression levels of 21 genes were determined by qRT-PCR using SYBR Green qRT-PCR Mix (Bio-Rad, Hercules, CA, USA) in a qTOWER^3^G Real-Time PCR Detection System (Analytik Jena AG, Jena, Germany) according to the manufacturer’s instructions. The RNA samples were the same as those used for RNA-Seq. First strand cDNA was synthesized from each sample total RNA with PrimeScript^TM^ RT reagent Kit with gDNA Eraser (Takara, Dalian, China) according to manufacturer’s protocols. The silkworm actin 3 (*BmActin3*, *BGIBMGA005576*), glyceraldehyde-3-phosphate dehydrogenase (*GAPDH*, *BGIBMGA007490*), and ribosomal protein L3 (*RpL3*, *BGIBMGA013567*) were selected as reference genes. All primers used for this study were shown in Supplementary Table S6. The qRT-PCR cycling parameters were as follows: 95 °C for 3 min, followed by 40 cycles of 95 °C for 10 s, and annealing at 60 °C for 30 s. All reactions were done in triplicate. The gene expression levels were normalized against the expression levels of the ribosomal protein L3 (*RpL3*). Additionally, the relative expression ratio of the target genes was analyzed using the 2^−ΔΔ*C*t^ method [94].

## 5. Conclusions

A total of 19,969 assembled transcripts were obtained from the integuments from wild-type Dazao and *sk* mutant at L5D2, a time point that represents an important stage of endocuticle generating in which cuticular protein genes were highly expressed and chitin was also synthesized in large quantities. These data therefore provide a reference for screening related genes for their effect on integument construction. Moreover, using comparative analyses, we detected eight cuticular protein genes, one chitinase gene, and one trehalase gene that may be potential candidate DEGs that are involved in exoskeleton shape formation. In addition, we identified a large number of antimicrobial peptide genes that are significantly downregulated in the *sk* mutant, indicating lower resistance than the wild-type Dazao. Furthermore, investigation of the dynamic mechanical properties of larval cuticle revealed that the cuticle of the *sk* mutant has a higher degree of stiffness and less damping oscillation compared to the wild-type, suggesting that the *sk* mutant has a lower capacity to adapt to its immediate environment. Integument formation, especially the endocuticle, require the cross-linking of cuticular proteins and chitin in the cuticle. If cuticular protein genes and genes or enzymes related to chitin synthesis and metabolism expression levels are insufficient, then correspondingly insufficient amounts of cuticular protein and chitin are produced in the cuticle. We, therefore, propose that the cuticular proteins and trehalase that are downregulated and the chitinase that is upregulated in the *sk* mutant may result in abnormally shaped exoskeletons. These findings improve our understanding of the molecular mechanisms underlying exoskeleton shape formation in the *sk* strain. The functions of the abovementioned genes will be verified in future studies.

## Figures and Tables

**Table 1:** GO functional enrichment analysis of DEGs between Dazao and *sk*.

GO ID	Gene Ontology Term	GeneRatio	BgRatio	*p* Value	FDR
	**Molecular Function**				
GO:0004553	Hydrolase activity, hydrolyzing *O*-glycosyl compounds	13/323	82/5764	0.56 × 10^−3^	0.04
GO:0008146	Sulfotransferase activity	6/323	20/5764	0.59 × 10^−3^	0.04
GO:0016782	Transferase activity, transferring sulfur-containing groups	6/323	21/5764	0.78 × 10^−3^	0.04
GO:0016798	Hydrolase activity, acting on glycosyl bonds	13/323	85/5764	0.80 × 10^−3^	0.04
GO:0022857	Transmembrane transporter activity	35/323	385/5764	0.27 × 10^−2^	0.11
GO:0016705	Oxidoreductase activity, acting on paired donors, with incorporation or reduction of molecular oxygen	13/323	99/5764	0.33 × 10^−2^	0.11
GO:0008061	Chitin binding	8/323	46/5764	0.36 × 10^−2^	0.11
GO:0005215	Transporter activity	38/323	438/5764	0.40 × 10^−2^	0.11
GO:0005506	Iron ion binding	12/323	101/5764	0.01	0.20
GO:0004559	α-mannosidase activity	4/323	17/5764	0.01	0.20
	**Cellular Component**				
GO:0005576	Extracellular region	20/118	144/1987	0.19 × 10^−3^	0.01
GO:0033178	Proton-transporting two-sector ATPase complex, catalytic domain	2/118	11/1987	0.14	1.00
GO:0030133	Transport vesicle	1/118	10/1987	0.46	1.00
GO:0030684	Preribosome	1/118	10/1987	0.46	1.00
GO:0030054	Cell junction	1/118	11/1987	0.49	1.00
GO:0098796	Membrane protein complex	5/118	78/1987	0.50	1.00
GO:0016469	Proton-transporting two-sector ATPase complex	2/118	28/1987	0.50	1.00
GO:0031012	Extracellular matrix	1/118	12/1987	0.52	1.00
GO:0098797	Plasma membrane protein complex	1/118	12/1987	0.52	1.00
GO:0005694	Chromosome	2/118	29/1987	0.52	1.00
	**Biological Process**				
GO:0009605	Response to external stimulus	9/231	10/3710	1.15 × 10^−10^	5.38 × 10^−9^
GO:0009607	Response to biotic stimulus	9/231	10/3710	1.15 × 10^−10^	5.38 × 10^−9^
GO:0009617	Response to bacterium	9/231	10/3710	1.15 × 10^−10^	5.38 × 10^−9^
GO:0042742	Defense response to bacterium	9/231	10/3710	1.15 × 10^−10^	5.38 × 10^−9^
GO:0043207	Response to external biotic stimulus	9/231	10/3710	1.15 × 10^−10^	5.38 × 10^−9^
GO:0051707	Response to other organism	9/231	10/3710	1.15 × 10^−10^	5.38 × 10^−9^
GO:0098542	Defense response to other organism	9/231	10/3710	1.15 × 10^−10^	5.38 × 10^−9^
GO:0006952	Defense response	9/231	12/3710	2.26 × 10^−9^	9.26 × 10^−8^
GO:0051704	Multi-organism process	9/231	14/3710	1.84 × 10^−8^	6.71 × 10^−7^
GO:0002376	Immune system process	8/231	16/3710	1.67 × 10^−6^	4.98 × 10^−5^
GO:0006955	Immune response	8/231	16/3710	1.67 × 10^−6^	4.98 × 10^−5^
GO:0015074	DNA integration	10/231	47/3710	0.48 × 10^−3^	0.01
GO:0006030	Chitin metabolic process	9/231	47/3710	0.20 × 10^−2^	0.04
GO:0006040	Amino sugar metabolic process	9/231	47/3710	0.20 × 10^−2^	0.04
GO:1901071	Glucosamine-containing compound metabolic process	9/231	47/3710	0.20 × 10^−2^	0.04

**GeneRatio.** The denominator represents the total number of DEGs with GO annotation, and the numerator represents the number of DEGs mapped to each GO term. BgRatio: The denominator represents the total number of reference genes with GO annotation, and the numerator represents the number of reference genes annotated in each GO term. FDR: *p*-value in hypergeometric test after correction.

**Table 2:** DEGs involved in chitin metabolic and chitin binding, defense and immune, hydrolase, transferase between wild-type and *sk* mutant.

Name	Gene ID	Dazao1 FPKM	Dazao2 FPKM	Dazao3 FPKM	*sk*1 FPKM	*sk*2 FPKM	*sk*3 FPKM	log2 FoldChange	FDR
**Chitin binding and chitin metabolic**									
Chitin binding peritrophin A domain	*novel.3284*	21.23	53.76	14.25	7.81	0	4.59	−2.81	0.02
Chitin binding peritrophin A domain	*novel.4995*	2197.11	810.02	2889.63	109.39	172.40	303.74	−3.33	3.38 × 10^−9^
Chitin binding peritrophin A domain	*novel.6300*	36.52	75.27	58.77	17.58	19.42	8.02	−1.92	0.40 × 10^−2^
Chitin binding peritrophin A domain	*novel.8828*	709.16	957.87	943.91	409.22	526.90	559.34	−0.80	0.02
Chitin binding peritrophin A domain	*novel.311*	0	0.90	4.45	16.60	20.64	12.61	3.23	0.02
Chitin binding peritrophin A domain	*novel.5689*	427.20	414.87	353.52	774.50	807.35	798.89	1.00	3.32 × 10^−5^
Chitin binding peritrophin A domain	*novel.6971*	907.04	1027.76	1040.09	1899.62	3166.25	1856.81	1.22	0.51 × 10^−3^
BmCPAP1-D	*BGIBMGA006382*	27.18	52.87	26.71	362.34	350.86	679.69	3.71	1.53 × 10^−16^
Chitinase	*BGIBMGA008709*	35.67	29.57	35.62	21.49	173.61	209.75	2.00	0.03
**Hydrolase**									
Glycosyl hydrolase family 1	*novel.2031*	13.59	10.75	16.92	3.91	1.21	1.15	−2.67	0.04
Glycosyl hydrolase family 1	*novel.12594*	107.01	114.69	73.02	48.83	32.78	33.24	−1.35	0.48 × 10^−2^
Glycosyl hydrolase family 1	*novel.12616*	124.00	19.71	1.78	0	0	0	−7.90	0.02
Glycosyl hydrolase family 31	*BGIBMGA013995*	86.63	116.49	103.30	51.76	40.06	46.99	−1.14	0.70 × 10^−2^
Glycosyl hydrolases family 38 C-terminal domain	*novel.1424*	189.39	150.54	199.47	55.67	74.06	30.95	−1.75	9.54 × 10^−5^
Glycosyl hydrolases family 38 N-terminal domain	*novel.15227*	9.34	5.38	8.01	0	0	0	−5.22	0.02
α-1,2-mannosidase	*BGIBMGA002426*	879.01	478.49	1114.00	144.55	122.62	338.12	−2.03	0.29 × 10^−3^
Mannosyl-oligosaccharide α-1,2-mannosidase IA	*BGIBMGA002486*	62.85	52.87	96.17	21.49	7.28	25.22	−1.96	0.29 × 10^−2^
Trehalase 1B	*BGIBMGA005665*	84.93	63.62	60.55	24.42	30.35	25.22	−1.39	0.42 × 10^−2^
β-glucosidase	*BGIBMGA010811*	0	0	0	4.88	9.71	6.88	5.46	0.01
Destabilase	*novel.6630*	16.14	16.13	20.48	82.04	110.48	61.89	2.27	1.97 × 10^−6^
α galactosidase A	*novel.8493*	255.64	443.54	332.15	1616.39	1016.16	1565.68	2.03	8.92 × 10^−9^
**Transferase**									
Sulfotransferase family	*novel.1630*	327.83	408.60	273.38	614.32	675.01	706.05	0.98	0.14 × 10^−2^
Sulfotransferase family	*novel.2592*	545.24	826.15	708.83	197.29	154.18	205.17	−1.90	3.15 × 10^−10^
Sulfotransferase domain	*novel.6336*	81.53	98.56	138.03	40.04	48.56	43.55	−1.27	0.59 × 10^−2^
Sulfotransferase domain	*novel.15388*	177.50	225.80	206.59	0.98	2.43	0	−7.52	4.53 × 10^−15^
Heparan-sulfate 6-*O*-sulfotransferase 2	*BGIBMGA007552*	32.27	17.92	36.51	5.86	6.07	12.61	−1.83	0.04
Amine sulfotransferase	*BGIBMGA010842*	2.55	2.69	2.67	13.67	34.00	8.02	2.81	0.02
**Antimicrobial peptides**									
Cecropin A	*BGIBMGA006280*	454.37	282.25	518.26	12.70	26.71	30.95	−4.17	3.68 × 10^−21^
Cecropin A	*BGIBMGA014285*	241.20	233.87	281.39	3.91	23.07	0	−4.82	0.34 × 10^−2^
Cecropin B	*BGIBMGA000023*	163.06	60.93	153.16	0	0	0	−9.27	4.63 × 10^−11^
Cecropin B	*BGIBMGA000036*	172.41	38.53	126.45	2.93	10.93	24.07	−3.16	0.28 × 10^−3^
Cecropin CBM2	*BGIBMGA000021*	44.16	8.06	35.62	0	0	0	−7.17	1.98 × 10^−5^
Cecropin-D	*BGIBMGA000017*	287.06	339.60	274.27	45.90	98.34	191.41	−1.43	0.03
Enbocin2	*BGIBMGA000039*	82.38	37.63	75.69	2.93	8.50	12.61	−3.05	1.74 × 10^−5^
Enbocin3	*BGIBMGA000018*	9.34	14.34	8.90	0	0	2.29	−3.87	0.04
Moricin-1	*BGIBMGA011495*	95.97	87.81	58.77	29.30	18.21	24.07	−1.75	0.45 × 10^−3^

## Supplementary Materials

Supplementary materials can be found at http://www.mdpi.com/1422-0067/19/10/3158/s1.

## Abbreviations

L5D3	Day 3 of the fifth instar
L5D2	Day 2 of the fifth instar
GO	Gene ontology
KEGG	Kyoto Encyclopedia of Genes and Genomes
CPAP	Cuticle Proteins Analogous to Peritrophin
ChtBD2	Peritrophin A-type chitin-binding domain
DEGs	Differentially-expressed genes
DMA	Dynamic mechanical analysis
DEGs	Differentially-expressed genes
PCA	Principal component analysis
FDR	Multiple hypotheses test the corrected *p* value
qRT-PCR	Quantitative real-time reverse transcription PCR
CBD	Chitin-binding domains
PM	Peritrophic membrane
PMPs	Peritrophic matrix proteins
