# Supplementary material for: Comparative Analysis of the Integument Transcriptomes between stick Mutant and Wild-Type Silkworms
Source: Int J Mol Sci. 2018 Oct 14;19(10):3158. doi: 10.3390/ijms19103158 (PMC6214029; doi:10.3390/ijms19103158)
Supplement: Supplementary file 1 [file ijms-19-03158-s001.zip › Supplementary Table 5 (english edits).docx]

**Supplementary Table 5.** DEGs involved in two significant KEGG pathways.

| Name | Gene ID | Dazao1 FPKM | Dazao2 FPKM | Dazao3 FPKM | *sk*1 FPKM | *sk*2 FPKM | *sk*3 FPKM | log2  Fold Change | FDR |
| --- | --- | --- | --- | --- | --- | --- | --- | --- | --- |
| **Fructose and mannose metabolism** | | | | | | | | | |
| Mitochondrial enolase superfamily member 1-like | *BGIBMGA**014323* | 2.55 | 0.90 | 0 | 266.63 | 560.89 | 55.02 | 7.98 | 4.66 × 10^–13^ |
| Aldo-keto reductase AKR2E4 isoform X1 | *BGIBMGA012831* | 6307.67 | 5909.41 | 6824.68 | 2177.97 | 3568.10 | 3463.76 | –1.05 | 0.15 × 10^–2^ |
| GDP-mannose 4,6 dehydratase | *BGIBMGA008433* | 67.09 | 71.68 | 121.11 | 219.75 | 145.69 | 353.02 | 1.47 | 0.72 × 10^–2^ |
| Zinc-binding dehydrogenase/ Alcohol dehydrogenase GroES-like domain | *novel.11049* | 2.55 | 0 | 1.78 | 160.17 | 122.62 | 1.146 | 6.03 | 0.01 |
| GDP-mannose 4,6 dehydratase | *BGIBMGA005687* | 107.86 | 85.12 | 116.65 | 183.61 | 171.18 | 223.51 | 0.90 | 0.02 |
| Mannose-6-phosphate isomerase | *BGIBMGA006473* | 25.48 | 17.92 | 17.81 | 63.48 | 29.14 | 72.21 | 1.43 | 0.04 |
| **Tyrosine metabolism** | | | | | | | | | |
| Major royal jelly protein | *novel.3420* | 125.69 | 207.88 | 130.01 | 453.17 | 392.14 | 410.33 | 1.44 | 2.72 × 10^–5^ |
| Major royal jelly protein | *novel.12441* | 90.87 | 99.46 | 67.68 | 202.17 | 284.09 | 189.12 | 1.39 | 0.22 × 10^–3^ |
| L-dopachrome tautomerase yellow-f-like | *novel.12443* | 1.70 | 3.58 | 0.89 | 12.70 | 21.85 | 32.09 | 3.43 | 0.13 × 10^–2^ |
| Yellow8 | *BGIBMGA**014026* | 11.04 | 8.96 | 4.45 | 0 | 0 | 0 | -5.32 | 0.01 |
| Macrophage migration inhibitory factor | *BGIBMGA**002087* | 935.92 | 431.00 | 1128.25 | 164.08 | 212.46 | 497.44 | -1.51 | 0.02 |
