# Supplementary material for: Comparative Analysis of the Integument Transcriptomes between stick Mutant and Wild-Type Silkworms
Source: Int J Mol Sci. 2018 Oct 14;19(10):3158. doi: 10.3390/ijms19103158 (PMC6214029; doi:10.3390/ijms19103158)
Supplement: Supplementary file 1 [file ijms-19-03158-s001.zip › Supplementary Table 6 (english edits).docx]

**Supplementary Table 6.** Primers used for qRT-PCR analysis.

| Primer name | Gene annotation | Forward primer sequences (5’-3’) | Reverse primer sequences (5’-3’) |
| --- | --- | --- | --- |
| BGIBMGA000419 | 3-hydroxyacyl-coa dehyrogenase | GCTGAAGGCATGCAAAGTTATA | TCTTTCTTGAGGAGTGACAGAC |
| BGIBMGA002940 | Putative alcohol dehydrogenase | GAAAGCAGAATGGCGGTAAAGG | CACAAGTGCCGTCTATTTCTTCATC |
| BGIBMGA006775 | Lebocin-3 precursor | CGTCCATTGAACATTCACATCA | CTCATCATAATGCCGCAATTCT |
| BGIBMGA014360 | Immune-related protein | GTCTTCTCTCAGCTCATCAACT | TAATTGGAGCAGACTGTACTCC |
| BGIBMGA002426 | Alpha 1,2-mannosidase | CCGGTGATCTTTATCCGAACTA | TTCCATCTTCTCTTCGATGACC |
| BGIBMGA007259 | Dicarbonyl L-xylulose reductase | AAATGGGTAGAAAAGTTTGGGC | CAAAGCAGGAATATTACCGCAT |
| BGIBMGA011087 | Prenyl-dependent CAAX metalloprotease | ACGGAGGATGAGATATTAGGGA | CATACATTCTAGAGACAGCCGT |
| BGIBMGA012968 | Bm8 interacting protein 2d-4 | AGTGTGAACTGGATTCTCAGTG | AGTTACCATTCCTCTTGTACCG |
| BGIBMGA000336 | Cuticular protein RR-1 motif 34 | GATCCTATACATACACAGCCCC | TGCTGGTAGTATTGACCTTTGT |
| BGIBMGA000346 | Cuticular protein RR-1 motif 23 | CGGTGCTGGTAGCCCTTTG | AATGAGGTTTTGGGTTGGAAGC |
| BGIBMGA003481 | Wing disc-specific protein | CGGACTAGCTTACTCGAAATCT | TAACCTCCTTTTGTGGGAAAAC |
| BGIBMGA008354 | Odorant binding protein | TGTAGCCGCTACTATTGAGAAG | CTTTGTTTGCAACGAAACAGTC |
| BGIBMGA003057 | Alpha amylase | ATACGTAAGCTGGGAAGATACG | CTACTGGCAGCCATGTTTTATC |
| BGIBMGA007601 | Delta-1-pyrroline-5-carboxylate dehydrogenase, mitochondrial | ATGACAAGCTCATGACAGAAGA | TCATTAGTACCTGACATACGGC |
| BGIBMGA007629 | Innexin 2 | TTTCTTTGTTATACCGCATGGC | GTCGTTTCCTTCAAACTTCTCC |
| BGIBMGA011718 | Neuropeptide-like 4C | CTTTGGTGTATGAGTACCCAGT | CGAGTAGGTGTAGGCAGAATAG |
| BGIBMGA000371 | Cuticular protein RR-1 motif 36 | TGATACTGCAGGAGAAACTGTT | GTAGCTAAGTACTGCAAAGCAC |
| BGIBMGA006662 | Cuticular protein glycine-rich 14 | CTACCAATCCGTCTCTTCTTCC | GTAACCAGAGATGCCAGAGTAG |
| BGIBMGA007285 | Ommochrome-binding protein | GAACTGACCGACATCAAAACAA | GCATTCGGAGTTGAAAAGTACA |
| BGIBMGA011862 | Protein arginine N-methyltransferase 7 | ACTTCTAGACTGATAAGCGACG | AGGACTCTTTATTGGACCTGTG |
| BGIBMGA000563 | Tyrosine hydroxylase | TTGATGCCCAAACACGC | TCGCAGGGTAAAGCCAGT |
| BGIBMGA005576 (BmActin3) | Cytoplasmic actin A3a1 | CCGTATGCGAAAGGAAATCA | TTGGAAGGTAGAGAGGGAGG |
| BGIBMGA007490 (GAPDH) | Glyceraldehyde-3-phosphate dehydrogenase | TTCATGCCACAACTGCTACA | AGTCAGCTTGCCATTAAGAG |
| BGIBMGA013567 (RPL3) | Ribosomal protein L3 | GAAGATGATCCGCTACTGT | TATCCTTTGCCCTTGGTG |
