# Supplementary material for: Comparative Analysis of the Integument Transcriptomes between stick Mutant and Wild-Type Silkworms
Source: Int J Mol Sci. 2018 Oct 14;19(10):3158. doi: 10.3390/ijms19103158 (PMC6214029; doi:10.3390/ijms19103158)
Supplement: Supplementary file 1 [file ijms-19-03158-s001.zip › Supplementary Table 1 (english edits).docx]

**Supplementary Table 1.** Assembly quality statistics of RNA-Seq libraries and mapping results.

|  | **Dazao1** | **Dazao2** | **Dazao3** | ***sk* 1** | ***sk* 2** | ***sk* 3** |
| --- | --- | --- | --- | --- | --- | --- |
| Raw reads | 33,319,522 | 30,619,179 | 36,306,278 | 29,760,402 | 32,365,793 | 29,340,753 |
| Clean reads | 32,482,554 | 30,066,688 | 35,220,034 | 29,022,685 | 31,610,204 | 28,270,732 |
| Clean bases (G) | 9.74 | 9.02 | 10.57 | 8.71 | 9.48 | 8.48 |
| Q20 | 97.29 | 94.78 | 97.15 | 96.32 | 96.35 | 97.32 |
| Q30 | 92.93 | 87.31 | 92.65 | 90.89 | 90.95 | 93.01 |
| GC content (%) | 49.77 | 49.81 | 50.07 | 50.18 | 50.38 | 50.01 |
| Total reads | 64,965,108 | 60,133,376 | 70,440,068 | 58,045,370 | 63,220,408 | 56,541,464 |
| Total mapped | 60,017,513 (92.38%) | 53,880,532 (89.60%) | 64,796,676 (91.99%) | 51,128,333 (88.08%) | 56,260,392 (88.99%) | 50,493,417 (89.30%) |
| Uniquely mapped | 58,300,414 (89.74%) | 52,185,432 (86.78%) | 62,750,586 (89.08%) | 49,383,492 (85.08%) | 54,636,685 (86.42%) | 49,323,047 (87.23%) |
| Multiple mapped | 1,717,099 (2.64%) | 1,695,100 (2.82%) | 2,046,090 (2.90%) | 1,744,841 (3.01%) | 1,623,707 (2.57%) | 1,170,370 (2.07%) |
| Exon | 6,531,626,146 (72.71%) | 5,718,583,272 (70.95%) | 7,286,111,400 (75.14%) | 5,684,925,385 (74.34%) | 6,281,899,201 (74.65%) | 567,9252,538 (75.20%) |
| Intron | 260,068,504 (2.90%) | 223,999,936 (2.78%) | 246,602,413 (2.54%) | 189,777,103 (2.48%) | 204,107,071 (2.43%) | 182,564,804 (2.42%) |
| Intergenic | 2,191,665,532 (24.40%) | 2,117,958,492 (26.28%) | 2,164,285,321 (22.32%) | 1,772,224,167 (23.18%) | 1,929,303,095 (22.93%) | 1,690,366,499 (22.38%) |
