# Supplementary material for: Comparative Analysis of the Integument Transcriptomes between stick Mutant and Wild-Type Silkworms
Source: Int J Mol Sci. 2018 Oct 14;19(10):3158. doi: 10.3390/ijms19103158 (PMC6214029; doi:10.3390/ijms19103158)
Supplement: Supplementary file 1 [file ijms-19-03158-s001.zip › Supplementary Table 4 (english edits).docx]

**Supplementary Table 4.** Enriched KEGG pathways in RNA_Seq.

| **Term** | **KEGG ID** | **DEGs with pathway annotation** | **All genes with pathway annotation** | ***P*-Value** | **FDR** | **gene ID** |
| --- | --- | --- | --- | --- | --- | --- |
| Fructose and mannose metabolism | bmor00051 | 6 | 20 | 8.82E-05 | 0.47×10-2 | BGIBMGA014323\|BGIBMGA012831  BGIBMGA008433\|novel.11049  BGIBMGA005687\|BGIBMGA006473 |
| Tyrosine metabolism | bmor00350 | 5 | 19 | 0.71×10-3 | 0.02 | novel.3420\|novel.12441  novel.12443\|BGIBMGA014026 BGIBMGA002087 |
| Pentose and glucuronate interconversions | bmor00040 | 5 | 35 | 0.01 | 0.21 | BGIBMGA000419\|BGIBMGA005443 BGIBMGA012831\|BGIBMGA007259 novel.11049 |
| N-Glycan biosynthesis | bmor00510 | 3 | 17 | 0.03 | 0.38 | BGIBMGA008394\|BGIBMGA002426  novel.6199 |
| Aminoacyl-tRNA biosynthesis | bmor00970 | 3 | 20 | 0.04 | 0.44 | novel.14740\|BGIBMGA007637  BGIBMGA014443 |
| Biosynthesis of unsaturated fatty acids | bmor01040 | 3 | 21 | 0.05 | 0.44 | BGIBMGA014550\|BGIBMGA006253 BGIBMGA001396 |
| Alanine, aspartate and glutamate metabolism | bmor00250 | 2 | 13 | 0.09 | 0.60 | novel.12821\|BGIBMGA007601 |
| Arginine and proline metabolism | bmor00330 | 2 | 13 | 0.09 | 0.60 | BGIBMGA007601\|BGIBMGA005812 |
| Amino sugar and nucleotide sugar metabolism | bmor00520 | 3 | 28 | 0.10 | 0.60 | BGIBMGA008433\|BGIBMGA005687 BGIBMGA006473 |
| TGF-beta signaling pathway | bmor04350 | 2 | 17 | 0.15 | 0.65 | BGIBMGA002340\|novel.4631 |
| Phototransduction - fly | bmor04745 | 2 | 17 | 0.15 | 0.65 | BGIBMGA008283\|novel.12816 |
| Drug metabolism - other enzymes | bmor00983 | 3 | 35 | 0.17 | 0.65 | BGIBMGA006965\|BGIBMGA005443 BGIBMGA008440 |
| Citrate cycle (TCA cycle) | bmor00020 | 2 | 20 | 0.19 | 0.65 | novel.13103\|novel.14950 |
| Fanconi anemia pathway | bmor03460 | 2 | 20 | 0.19 | 0.65 | BGIBMGA008315\|BGIBMGA009157 |
| Fatty acid metabolism | bmor01212 | 3 | 38 | 0.20 | 0.65 | BGIBMGA014550\|BGIBMGA006253  BGIBMGA001396 |
| Longevity regulating pathway - multiple species | bmor04213 | 3 | 38 | 0.20 | 0.65 | BGIBMGA004541\|BGIBMGA014536BGIBMGA004540 |
| Ribosome biogenesis in eukaryotes | bmor03008 | 3 | 41 | 0.23 | 0.70 | BGIBMGA005597\|BGIBMGA005552  BGIBMGA006056 |
| mRNA surveillance pathway | bmor03015 | 3 | 42 | 0.24 | 0.70 | novel.14519\|BGIBMGA003125  BGIBMGA002340 |
| Pyrimidine metabolism | bmor00240 | 3 | 46 | 0.29 | 0.70 | BGIBMGA006965\|BGIBMGA006292  BGIBMGA004166 |
| Drug metabolism - cytochrome P450 | bmor00982 | 2 | 27 | 0.30 | 0.70 | BGIBMGA005443\|BGIBMGA002211 |
| beta-Alanine metabolism | bmor00410 | 1 | 10 | 0.34 | 0.70 | BGIBMGA006965 |
| Metabolism of xenobiotics by cytochrome P450 | bmor00980 | 2 | 30 | 0.35 | 0.70 | BGIBMGA005443\|BGIBMGA002211 |
| Galactose metabolism | bmor00052 | 1 | 11 | 0.37 | 0.70 | BGIBMGA012831 |
| Fatty acid elongation | bmor00062 | 1 | 11 | 0.37 | 0.70 | BGIBMGA001396 |
| Starch and sucrose metabolism | bmor00500 | 1 | 11 | 0.37 | 0.70 | BGIBMGA005665 |
| Notch signaling pathway | bmor04330 | 1 | 11 | 0.37 | 0.70 | BGIBMGA010195 |
| Protein processing in endoplasmic reticulum | bmor04141 | 4 | 78 | 0.39 | 0.70 | BGIBMGA002426\|BGIBMGA004541  BGIBMGA014536\|BGIBMGA004540 |
| Hippo signaling pathway - fly | bmor04391 | 2 | 34 | 0.40 | 0.70 | novel.12816\|BGIBMGA002340 |
| Wnt signaling pathway | bmor04310 | 2 | 36 | 0.43 | 0.70 | BGIBMGA008283\|novel.4631 |
| Purine metabolism | bmor00230 | 4 | 84 | 0.45 | 0.70 | BGIBMGA006292\|novel.12821  BGIBMGA000807\|BGIBMGA008440 |
| Phagosome | bmor04145 | 2 | 37 | 0.45 | 0.70 | BGIBMGA003296\|novel.12816 |
| Terpenoid backbone biosynthesis | bmor00900 | 1 | 15 | 0.46 | 0.70 | BGIBMGA011087 |
| ECM-receptor interaction | bmor04512 | 1 | 15 | 0.46 | 0.70 | BGIBMGA002018 |
| AGE-RAGE signaling pathway in diabetic complications | bmor04933 | 1 | 15 | 0.46 | 0.70 | BGIBMGA008283 |
| Endocytosis | bmor04144 | 3 | 62 | 0.46 | 0.70 | BGIBMGA014536\|BGIBMGA007010  BGIBMGA011157 |
| Pentose phosphate pathway | bmor00030 | 1 | 18 | 0.53 | 0.74 | BGIBMGA013916 |
| Insect hormone biosynthesis | bmor00981 | 1 | 18 | 0.53 | 0.74 | BGIBMGA009211 |
| Spliceosome | bmor03040 | 3 | 68 | 0.53 | 0.74 | novel.11183\|BGIBMGA014536  BGIBMGA013891 |
| Retinol metabolism | bmor00830 | 1 | 21 | 0.58 | 0.76 | BGIBMGA005443 |
| Glutathione metabolism | bmor00480 | 1 | 22 | 0.60 | 0.76 | BGIBMGA002211 |
| RNA polymerase | bmor03020 | 1 | 22 | 0.60 | 0.76 | BGIBMGA006292 |
| RNA transport | bmor03013 | 3 | 76 | 0.61 | 0.76 | BGIBMGA003125\|novel.4267  novel.13009 |
| Folate biosynthesis | bmor00790 | 1 | 24 | 0.63 | 0.76 | BGIBMGA012831 |
| Toll and Imd signaling pathway | bmor04624 | 1 | 24 | 0.63 | 0.76 | novel.1072 |
| Carbon metabolism | bmor01200 | 2 | 56 | 0.67 | 0.77 | BGIBMGA013916\|novel.7525 |
| Ascorbate and aldarate metabolism | bmor00053 | 1 | 27 | 0.68 | 0.77 | BGIBMGA005443 |
| RNA degradation | bmor03018 | 1 | 29 | 0.70 | 0.77 | novel.14894 |
| Peroxisome | bmor04146 | 2 | 60 | 0.71 | 0.77 | BGIBMGA002186\|BGIBMGA008440 |
| Porphyrin and chlorophyll metabolism | bmor00860 | 1 | 30 | 0.71 | 0.77 | BGIBMGA005443 |
| Glycerolipid metabolism | bmor00561 | 1 | 32 | 0.74 | 0.77 | BGIBMGA012831 |
| Inositol phosphate metabolism | bmor00562 | 1 | 32 | 0.74 | 0.77 | BGIBMGA008283 |
| Phosphatidylinositol signaling system | bmor04070 | 1 | 34 | 0.76 | 0.77 | BGIBMGA008283 |
| Lysosome | bmor04142 | 1 | 62 | 0.93 | 0.93 | novel.4358 |
